# Association between type 2 diabetes mellitus, biochemical factors and UCSNP-43 polymorphisms of CALPIN-10 gene in patients with atherosclerosis of coronary artery disease in Southern Iran population

**DOI:** 10.15171/jcvtr.2016.03

**Published:** 2016-03-14

**Authors:** Sara Senemar, Mohammad Reza Edraki, Samane Toosi

**Affiliations:** ^1^Human Genetics Research Group, Iranian Academic Center for Education & Research (ACECR), Fars Branch, Shiraz, Iran; ^2^Institute for Pediatric Cardiologist, Shiraz University of Medical Sciences, Shiraz, Iran

**Keywords:** SNP-43 Polymorphism, CALPIN-10, Coronary Artery Disease, Atherosclerosis, Lipid Profile, Diabete

## Abstract

***Introduction:*** Genetic variations in the calpain 10 gene (CALPIN-10), single nucleotide polymorphisms-43 (SNP-43), have increased the risk of type 2 diabete mellitus (T2DM) and coronary artery disease (CAD).

***Methods:*** We studied the control and CAD groups for association of association of SNP-43 in the CALPIN-10 gene with T2DM and other risk factors of its complications. Overall, we examined 452 individuals, 224 patients with CAD and 228 healthy subjects for CAD in Iranian population. All the subjects were genotyped for the CALPIN-10, SNP-43 by polymorphism chain reaction (PCR) and restriction fragment length polymorphism (RFLP) methods, using biochemical methods to detect fasting glucose and other biochemical factors in the blood sample. We assessed frequencies of SNP-43 alleles between CAD and normal population groups.

***Results:*** In CAD patients, the GG allele was significantly associated with T2DM and GG allele was causing high level of glucose. But in control group, there was no relationship between them. Between clinical and biochemical risk factors with different genotypes there was no significant difference in the compared group.

***Conclusion:*** The results of our study suggest no significant association between SNP-43 and the risk of T2DM. In other words, CALPIN-10 did not show a major diabetes gene pool capacity in normal southern Iranian population.

## Introduction

Atherosclerosis is a major cause of death among adults in developed and developing countries. Atherosclerosis will lead to blocked coronary artery. Premature coronary heart disease (PCAD) is influenced by some genetic, environmental and metabolic disease factors. Metabolic syndrome is caused by some factors, the most important of which is diabetes mellitus (DM).^1^

DM is the background of coronary artery disease (CAD).^2^ Diabetes is an autoimmune disease and it emergences from defects in insulin secretion and action. Decreasing insulin hormone causes diabetic people to get energy from food.^3^ It is a group of metabolic diseases in which there are high blood sugar levels over a long period of time.^4^ In 2014, it was estimated that 387 million people have diabetes worldwide.^5^ 8.3% of the adult population have DM and men and women are equal in terms of the rate of the disease.^6,7^ More than 4 million Iranian adults have diabetes and it has increased by 35% over the 2005-2011.^8^ In Iranian adults, a striking prevalence of DM was 11.4% recorded and 14.6% of Iranian adults were diagnosed with impaired fasting glucose. It is significantly lower than the rates observed in the United States (14.6%); the prevalence of diabetes in Iran is significantly higher than its neighboring countries, such as Pakistan^9^ (6.7%) and Turkey (7.2),^10^ but it falls below the incidence of diabetes in Arab countries.^11^ Horikawa et al estimated the population-attributable risk of diabetes at 14%, whereas this risk among Caucasians was estimated only 4%.^12^

Although the physical activity and changes in lifestyle and diet can reduce the complications of type 2 diabetes, recognizing the genetic risk factors is very important for its prevention and treatment. Some genes are the basis for DM. One of the genes is calcium-activate neutral protease-10 (CALPIN-10). In addition, CALPIN-10 has an important role in proliferation and migration of vascular smooth muscle cell; platelet aggregation, degranulation, and spreading are the other factors.^13,14^ This gene is a member of the calpain family. CALPIN-10 gene located at 2q37 and spans 31 kb and contains 15 exons, constituted by 672 amino acids; it is in the third intron region.^15^ CALPIN-10 gene was the first diabetes gene identified by cloning and encodes the calpain-10 protein.^12^ Calpain-10 protein is involved in the secreting pancreatic islands and action of insulin, and is associated with adipocytes.

This gene encodes a nonlysosomal cysteine protease and ‘contributes to’ atherosclerosis and is associated with several metabolic syndromes, for example diabetes, BMI, lipid profile (plasma cholesterol and triglyceride concentrations); it finally leads to atherosclerosis of CAD.^16,17^

One of the variation of CALPIN-10 is SNP-43 that this variant is the result of converting amino acids (arginine into guanine). SNP-43 is an A-to-G variant in intron 3 of the CALPIN-10 gene and is identified as a possible type 2 diabetes.^18^ Baier et al^19^ showed that the A allele had little promoter activity than the G allele and G/G genotype. It seems that it makes an association between the G/A and A/A genotype and reduced CALPAIN-10 mRNA expression. The G allele (G/G genotype) is more associated with higher blood glucose than A allele (G/A and A/A).^17^

Although many studies on the correlation between CALPIN-10, SNP-43 G>A gene polymorphism and DM have been performed so far, existence of this relationship is not clear to researchers. A significant correlation was observed in African-Americans,^20^ Caucasians, Hispanics,^21^ Japanese Americans,^22^ and Caucasians from the United States,^23^ France,^24^ Finland,^21,25-27^ and the United kingdom.^28^

Variation in the CALPAIN-10 gene has been linked to 2.8-fold increased risk for type 2 diabetes in Mexican-Americans and some European populations, but in some communities conflicting results have been produced, for example Pima Indian and South Indian. In the present project, we plan to examine this relationship between SNP-43, diabetes and the risk of early form of PCAD in Iran. To this aim, we involved the patients and controls of CAD and aimed to determine the relationship of CALPAIN-10 SNP-43 G/A gene polymorphism and T2DM in the Iranian population. Also, we collected the information about the patients, including age, sex, race, personal and family history of diabetes, fasting blood sugar, and hyperlipidemia profile (total cholesterol, high-density lipoprotein [HDL] cholesterol, and triglycerides). We used a modified version of the questionnaire developed by Baecke et al.^29^

## Material and methods

### Study population

This is a case-control study on 452 subjects with an age range of 22-81 and mean age of 52.58±12.88 years. They had undergone coronary angiography. Positive angiography has been explained as coronary diameter decrease greater than 50% as described by a cardiologist while control subjects had fewer than 30% stenosis in all major vessels. A total of 224 patients were diagnosed with positive angiographic stenosis (cases) and 228 with negative angiographic stenosis (controls). We matched them for age, sex and ethnicity. We excluded the patients with a history of using drugs for blood pressure, blood sugar, those were fat, and those who smoked more than one pack a day (for professionals). The questionnaire was filled out by two trained interviewers about cardiovascular disease such as family history, cigarette smoking, age, and hypertension.

### Biomedical measurement

Ten milliliters of the whole blood samples was taken from the brachial vein of each subject with suspected CAD in the early morning fasting (12 hours). One of two test tubes contained EDTA (about 5 mL) and the other without EDTA (about 5 mL) and in 1 hour were transferred to the medical lab. To measure the glucose, triglyceride (TG), high-density lipoprotein (HDL), low-density lipoprotein (LDL), and cholesterol concentration, we used samples of blood poured into tubes without anticoagulant. Standard enzymatic assay (Pars Azemoon, Diagnostic Kit) was used to measure the biochemical parameters. High standards for blood pressure, sugar and fat were calculated as follows: hypertension (>130/85 mm Hg), HDL low levels (<40 mg/dl in men and <50 mg/dl in women), high triglyceride levels (>150 mg/dl), LDL high levels (>160 mg/dl) and hyperglycemia (>100 mg/dl), and diabetes according to the criteria of the American diabetes Association (ADA) (blood glucose level >126 mg/dl ).

Genomic DNA was extracted from 5 ml blood samples with EDTA by salting out method. The analysis of the SNP-43 polymorphism in the CALPIN-10 gene was performed by polymorphism chain reaction (PCR) coupled restriction fragment length polymorphism (RFLP) method.^30^ A PCR was performed with 20 ng DNA using the following primers:

Sense 5’ GCTGGCTGGTGACATCAGTG3’

Antisense 5’ TCAGGTTCCATCTTTCTGCCAG3

The resulting 144-bp product was digested with 1 ml Nsi (Cinnagene, Iran) at 37°C for overnight (15-18 hours). We identified the digested products with electro­phoresis on 3.5% agarose gel followed by ethidium-bro­mide staining and visualized by UV light to identify SNPs-43 and visualized by ultraviolet light to determine the SNPs. The percentage of genotype(s) for all the three loci performed under co-dominant, dominant and recessive models was 134 bp for allele 1 (G) and 152 bp for allele 2 (A).

### Statistical analysis

Statistical analysis was carried out using SPSS version 11.5 for Windows (SPSS Inc, Chicago, IL, USA). *P*<0.05 was considered as statistically significant. Comparisons of frequencies between qualitative variables were carried out by chi-square test. Also, to assess the Hardy-Weinberg equilibrium (HWE), a logistic regression model was fitted to examine the independent impact of different clinical and biochemical factors on CAD. To determining distribution alleles in the groups (case and control), we used chi-square test. The frequencies of different genotypes were obtained; then allele frequencies were calculated in cases and controls and then tabulated. The normality of distributing the investigated variables was assessed using the Kolmogorov–Smirnov criterion. Since TG, Total cholesterol (TC)and HDL were not normally distributed, the results of these variables were assessed using non-parametric tests (Mann-Whitney and Kruskal-Wallis). For normal parameters, such as fasting blood sugar (FBS) and LDL, parametric method was used (*t* test and ANOVA). Respective odds ratios (OR) were calculated for an unadjusted analysis and for an adjusted model which had been controlled for parameters that may contribute to the risk of CAD, such as CALPIN-10 genotype, smoking statue, diabetes, hypertension, lipid profile, and familial history.

## Results

Clinical and baseline characteristics and of cases and controls are summarized in Table 1.

**Table 1:** Distribution of CALPIN-10, SNP-43 gene polymorphism genotype among case and control groups

**Genotype**	**Case ** **No. (%)**	**Control ** **No. (%)**	*P* ** value**
GG	138 (67)	95 (67.9)	0.477
GA	51 (27.8)	29 (20.7‏)	
AA	17 (8.3)	16 (11.4‏)	
Total	206	140	

CALPIN-10 mutation frequency is presented in Table 1 for the case and control groups. The frequency of genotypes GG, GA and AA patients and healthy individuals was 66.9%, 24.75% and 8.25% (for patients) and 67.9%, 20.7%, 11.4% (for normal people). There was no significant difference between two groups regarding genotype and allele frequencies. The genotype frequencies of G/G, A/G, and A/A were 67.3%, 23.1%, and 9.5%, respectively in the whole population and not according to Hardy-Weinberg equilibrium (χ²) (Table 2).

**Table 2:** Hardy-Weinberg equilibrium test of CALPIN-10 and SNP-43

**Genotype count**	**Observed count**	**Expected frequency**	**Genotype count**	**Allele**	**Observed**	**χ²**
GG	233	216	67.3	G	0.79	11.982
GA	80	115	23.1	A	0.21	
AA	33	15	9.5			

Table 3 shows distribution of biochemical risk factors studied between CALPIN-10 genotypes in the total subjects. As shown in  Table 3, none of the risk factors and CALPIN-10 genotypes in all subjects showed a significant relationship.

**Table 3:** Distribution of clinical and biochemical risk factors between different genotypes of CALPIN-10, SNP-43 in total research

**Risk factor**	**GG** **Mean (±SD)**	**GA** **Mean (±SD)**	**AA** **Mean (±SD)**	**P value**
FBS (mg/dl)	113.36 (66.85)	‏(63.66‏) 109.45	109.85 (76.68)	‏0.664^a^
TG (mg/dl)	137.88 (74.03)	126.19 (70.09)	‏(81.86‏) 137.91	‏0.063^b^
Cholesterol (mg/dl)	‏(64.27) 134.2	‏(69.63) 144.42	‏(73.18‏) 146.18	‏0.118^b^
HDL (mg/dl)	‏(37.77‏) 36.24	‏(15.88) 32.05	‏(15.31) 29.91	‏0.422^b^
LDL (mg/dl)	‏(45.55‏) 80.69	‏(44.88) 85.74	‏(47.71‏) 91.58	‏0.693^a^

Abbreviations: FBS, fasting blood sugar; HDL, high density lipoprotein; LDL, low density lipoprotein; mg/dl, milligram per deciliter; SD, standard deviation; TC, total cholesterol; TG, triglyceride. ^a^Calculated by nonparametric methods (Kruskal-Wallis ); ^b^Calculated by parametric methods (ANOVA).

A significant relationship was seen between the high levels of blood glucose (diabetes) in patients and genotype CALPIN-10 (Table 4).

**Table 4:** Distribution of qualitative information based on clinical and biochemical case group

**Risk factors**	**GG (%)**	**GA (%)**	**AA (%)**	***P *** ** value**
FBS (>100 mg/dl)	121 (58.8)	64 (31)	21 (10.2)	0.045
TG (>200 mg/dl)	151 (73.3)	37 (18)	18 (8.7)	‏0.567
Cholesterol (>200 mg/dl)	103 (50)	68 (33)	35 (17)	‏0.650
HDL (<35 mg/dl)	140 (68)	43 (21)	23 (11)	‏0.311
LDL (>130 mg/dl)	30 (14.5)	118 (57.3)	58 (28.2)	‏0.100
Hypertension	52 (25)	101 (49)	53 (26)	0.500
Smoking	142 (68.9)	49 (23.8)	15 (7.3)	0.873
Family history	135 (65.5)	54 (26.5)	17 (8)	0.885

Abbreviations: FBS, fasting blood sugar; HDL, high density lipoprotein; LDL, low density lipoprotein; mg/dl, milligram per deciliter; TG, triglyceride.

Between clinical and biochemical risk factors with different genotypes there was no significant relationship in the control group (Table 5).

**Table 5:** Distribution of qualitative information based on clinical and biochemical control group

**Risk factors**	**GG (%)**	**GA (%)**	**AA (%)**	***P *** **value**
FBS (>100 mg/dl)	139 (67.5)	44 (21.3)	23 (11.2)	0.904
TG (>200 mg/dl)	141 (68.5)	33 (16)	32 (15.5)	0.641
Cholesterol (>200 mg/dl)	70 (34)	68 (33.3)	68 (33.3)	0.337
HDL (<35 mg/dl)	133 (64.6)	53 (25.7)	20 (9.7)	0.652
LDL (>130 mg/dl)	120 (58.3)	32 (15.5)	54 (26.2)	0.289
Hypertension	138 (66.9)	48 (23.3)	21 (10.2)	0.851
Smoking	119 (57.8)	62 (30)	25 (122)	0.190
Family history	136 (66.1)	35 (16.9)	35 (16.9)	0.250

Abbreviations: FBS, fasting blood sugar; HDL, high density lipoprotein; LDL, low density lipoprotein; mg/dl, milligram per deciliter; TG, triglyceride.

There was a significant correlation in the level of clinical characteristics (FBS, TG, cholesterol, and HDL) between the control and patient groups (Table 6).

**Table 6:** Demographic and clinical characteristics in the control and patient groups

**Risk factors**	**Control Mean (±SD)**	**Case Mean (±SD)**	***P *** ** value**
FBS (> 100 mg/dl)	115.09 (70.220)	124.57 (59.255)	0.004
TG (>200 mg/dl)	152.83 (121.706)	154.45 (126.217)	0.049
Cholesterol (>200 mg/dl)	159.18 (48.404)	155.84 (42.696)	0.005
HDL (<35 mg/dl)	40.23 (17.865)	35.70 (10.437)	0.000
LDL (>130 mg/dl)	94.27 (18.643)	97.38 (38.502)	0.053

Abbreviations: FBS, fasting blood sugar; HDL, high density lipoprotein; LDL, low density lipoprotein; mg/dl, milligram per deciliter; TG, triglyceride.

## Discussion

Coronary heart disease is a major cause of mortality and morbidity in Middle East populations. A few convincing functional researches supported that some genes affect atherosclerosis and diabetes. On the other hand, CAD and T2DM have the same genetic backgrounds. They mostly happen with each other, and some records suggest this relation. Now, several genes recognized which influence diabetes and atherosclerosis.

Research on genetic factors of CAD is being conducted in Iranian populations. In most studies, it is shown that hereditary susceptibility explains about 50% of predisposition to CAD. One of the reasons of CAD is atherosclerotic plaque and this stenosis is caused by high blood sugar levels.

So far, many experimental studies have suggested a pathogenetic role for CALPIN-10 in various complications of CAD and diabete. Calpain-10 protein (intracellular Ca²^+^ dependent cysteine protease) is the risk factor of increasing blood sugar but this relevance has not been confirmed by many other researchers. In other words, the role of this polymorphism in development of diabetes in different populations is still controversial.

A gene sequence of a G/G combination at this sequence is the abnormal gene sequence polymorphism, and the normal sequence is the A/G combination. This research provided the opportunity to look at the effect of this genetic factor (SNP-43 polymorphisms) in T2DM risk on coronary artery patients potential interactions of extrinsic factors that are known to influence T2DM risk with the SNP-43 polymorphisms in coronary artery patients. We studied about SNP-43 polymorphism in controls and patients with coronary heart disease and correlation between SNP-43, G>A gene polymorphism and diabetes.

In this study, we showed G allele of CALPIN-10, SNP43 gene polymorphism did not increase the susceptibility to T2DM in the control groups of Iranian population (our data confirmed that the GG allele genotype slightly raised the fasting glucose level in the control group), but in the CAD patients, SNP-43 was significantly associated with T2DM.

There seems that not being a G/G polymorphism leads to high blood sugar levels in normal Iranian population but the correlation between gene polymorphism and CAD disease in patients is caused by high mean blood sugar (diabetes) as a risk factor for heart disease.

Our results support the idea that most of the genetic markers for type 2 diabetes (such as SNP-43) and metabolic syndrome are not independent pathogenic agents, but the markers associated with unhealthy environmental factors (high body mass index [BMI], unhealthy diet, bad lifestyle) can predispose one to diabetes. In other words, it does not seem that any relationship the UCSNP-43 polymorphism alone with diabetes involves impairment of insulin secretion in Iranian population. Similarly, this SNP-43 polymorphism was not associated with T2DM in recent studies in the United Kingdom,^30^ Pima Indians, German Caucasians,^31^ and Caucasian population^32^ in the south eastern areas.

Also, our results are consistent with the observations. The similar result might be due to the type of studies or correlation of polymorphisms between race and geographical location. The Iranian population is Caucasian and in this respect, genetic polymorphism of Iranian population is similarly close to other countries with the same race. Probably, we can offer this gene as a good marker for anthropological studies.

In another study on Northern Iranian population, the results showed that among the population of Azerbaijan (North Iran) G allele increased the risk of type 2 diabetes compared with allele A in diabetic patients. Also, this result is in the same line with those of Northern European, South Indians, and African-Americans (Table 7).^12,33,34^

**Table 7:** The allele frequencies of SNP-43 polymorphisms in CALPIN-10 gene in the earth’s population

**Continent and region**	**Populations**	**SNP-43 Alleles**	
		**G**	**A**
Africa	Baik (N=138)	‏0.91	‏0.09
	Mbuti (N=70)	‏0.97	‏0.03
Europe	Europeans (N=180)	‏0.80	‏0.20
	Danes (N=98)	‏0.63	‏0.37
	Druzel (N=126)	‏0.90	‏0.10
Asia	Chinese (N=110)	‏0.97	‏0.03
	Japan (N=72)	‏0.87	‏0.13
	Yakut (N=72)	‏0.85	‏0.15
Pacific Islands	Nasioi (N=44)	‏0.90	‏0.10
North and South America	Maya (N=94)	‏0.69	‏0.31
	Surui (N=92)	‏0.73	‏0.27
Iran	Eastern Azerbaijan province (N=202)	‏0.92	‏0.08
	Fars province (N=452)	‏0.79	‏0.21

In the past, some studies used the case-control method and others used the cross-sectional method. One of the strengths in this case-control study is the strong solidarity and in the present research we used the case-control method.

There are possible explanations to reconcile our findings with other studies inconsistent results as below:

First, the main reason for this difference is probably the ethnic variability and different nature of the genetic predisposing populations. Second, this research focused on a population with normal coronary artery rather than random normal population. Third, this study was conducted on a population that had no HWE.

Other findings of this project is investigating the relationship between SNP-43 and clinical characteristic factors. In the control and CAD patients groups, our data confirmed that no significant relationship existed among UCSNP-43 and hyperlipidemia, smoking, hypertension and family history, but in the patients group the level of this biochemical factor was higher than normal population. So these extrinsic risk factors do not play a major role in G/G homozygosity with CAD. Another research has shown the relationship between this gene and the high level of cholesterol, Triglyceride free fatty acid.^30^

Therefore, to understand the relationship and interaction between genes and the metabolic syndrome (e.g., diabetes), we should not overlook the role of nutrition and other factors.

High blood sugar was a strong risk factor for CAD in the south of Iran. One of the strengths in this study was the use of a case-control design.

Hyperlipidemia profile especially HDL is a strong factor in coronary disease in Iranian population. Briefly, we showed that this variation of CALPIN-10 was separately accompanied with clinical atherosclerosis of T2DM.

In the present study, We managed to genotype 452 families with several generations of conflict with coronary heart disease in Southern Iranian descent. We showed CALPIN-10, SNP-43 contributes to lead to coronary artery atherosclerosis in Iranian population diagnosed with atherosclerosis and insulin resistance but according to the survey results, G allele of SNP-43 CALPIN-10 polymorphism is not a risk factor for type 2 diabetes in the normal population of southern Iran. This increased risk in our families, which were ascertained to a strong history of T2DM.^35^

## Acknowledgements

The authors would like to thank the Center for Development of Clinical Research of Nemazee Hospital and Dr. Nasrin Shokrpour for editorial assistance.

## Ethical issues

The study was approved by the Ethics Committee of Shiraz University of Medical Sciences.

## Competing interests

Authors declare no conflict of interest in this study.
